# Experimental Characterization and Modeling of 3D Printed Continuous Carbon Fibers Composites with Different Fiber Orientation Produced by FFF Process

**DOI:** 10.3390/polym14030426

**Published:** 2022-01-21

**Authors:** Federico Lupone, Elisa Padovano, Cinzia Venezia, Claudio Badini

**Affiliations:** Department of Applied Science and Technology, Politecnico di Torino, Corso Duca degli Abruzzi 24, 10129 Torino, Italy; federico.lupone@polito.it (F.L.); cinzia.venezia@studenti.polito.it (C.V.); claudio.badini@polito.it (C.B.)

**Keywords:** continuous carbon fibers polymer composites, Fused Filament Fabrication, microstructure, tensile and failure behavior, rule of mixture, Volume Average Stiffness, Classical Laminate Theory

## Abstract

The development of 3D printed composites showing increased stiffness and strength thanks to the use of continuous carbon fibers has offered new prospects for Fused Filament Fabrication (FFF) technique. This work aims to investigate the microstructure and mechanical properties of 3D printed CCF/PA composites with various layups, and also to apply predictive models. The mechanical properties of the printed parts were directly related to the adopted laminate layup as well as to the microstructure and defects induced by the FFF process. The highest stiffness and strength were reported for longitudinal composites, where the fibers are unidirectionally aligned in the loading direction. In addition, it was found that the reduction in tensile properties obtained for cross-ply and quasi-isotropic laminate layups can be described by using the Angle Minus Longitudinal. A step-like failure with extensive fibers breakage and pull-out was observed for the longitudinal composites. By contrast, the rupture mode of the quasi-isotropic laminates mainly exhibited debonding between beads. Moreover, the predictions obtained using the Volume Average Stiffness method and Classical Laminate Theory were in good agreement with the tensile test results. This work could help engineers to design complex laminates with specific mechanical requirements by tailoring the orientation of continuous carbon fibers.

## 1. Introduction

Additive manufacturing (AM), also known as 3D printing, has aroused increasing interest in recent years since it offers important advantages over traditional fabrication methods such as high geometric complexity without needing molds, providing product customization and resulting in less wastage of raw materials [1].

Fused Filament Fabrication (FFF) technique is one of the most frequently employed AM processes due to a cost-effective and simple operation and availability of feedstock materials. In the FFF process, a three-dimensional (3D) object is produced directly from computer aided design (CAD) files by extrusion of a thermoplastic polymer thread through a heated nozzle. The molten filament is deposited onto the print bed in a raster pattern to create each layer, and the final 3D part is fabricated bottom up one layer at a time. Common feedstock materials include thermoplastic commodities (e.g., Poly Lactic Acid and Acrylonitrile Butadiene Styrene) and engineering polymers (e.g., Polyamides, Polycarbonate and others) [2]. However, lack of strength, stiffness and structural integrity due to polymers’ inherent low mechanical properties have restricted the application of FFF to prototypes and small end-use functional parts [2].

In order to overcome these limitations, short fiber reinforced polymers were extensively studied. In fact, carbon fibers exhibit an excellent combination of properties that allow their use in applications where lightweight, strength and/or specific functionalities (e.g., structural health monitoring sensors [3], energy storage [3,4], filtration or thermal insulation [5]) are required. Several authors [6,7,8,9,10] reported improvements on mechanical properties as well as dimensional accuracy of FFF printed polymeric parts by adding short carbon fibers to the matrix. Nevertheless, limitations in fiber length and volume fraction, and the increase in void content due to processing issues largely restrict the maximum mechanical properties achievable with short fiber composites that remain still inferior when compared to conventional composites [11,12,13]. 

In the hope of circumventing these drawbacks, great efforts have been carried out in adapting the FFF process to produce continuous carbon fiber (CF) reinforced polymer composites [13,14]. The company MarkForged^®^ first developed 3D printers capable of embedding continuous fibers directly in the thermoplastic parts using two separate printing heads for the neat polymer and the pre-impregnated reinforcing filaments, respectively [14,15,16]. The related technique was referred to as Continuous Filament Fabrication (CFF). In the last few years, extensive investigations have been performed in order to evaluate the microstructure and mechanical behavior of continuous carbon fiber reinforced polyamide (CFRPA) composites manufactured using FFF technique. Many authors found that process-induced defects, such as high void content, poor interlayer bonding and inhomogeneous fibers distribution, has a high impact on interlaminar shear strength and failure behavior of the printed objects [17,18,19,20,21]. However, a remarkable improvement in the mechanical properties has been reported for various load cases: tension [16,17,18,19,20,22], compression [18,20,23,24], bending [24,25,26], fatigue [27], creep [27] and impact [28]. The effect of build orientation, fiber content and infill pattern (i.e., isotropic or concentric fiber orientation inside a single layer) on the mechanical behavior of the 3D printed composites was also studied [15,21,29,30].

It is well known that the fibers display the uppermost beneficial effect on strength and stiffness when the load is applied in the fiber axial direction, while they exert only a negligible effect in the transverse direction. For this reason, conventional composites are designed by changing the fiber orientation in each ply to obtain a compromise between the improvement of mechanical performances and a suitable degree of isotropy. A similar strategy could be adopted in the case of 3D printed composites as well.

It was observed that 3D printed composites with fibers aligned in the loading direction showed the highest performances, while the transverse properties are significantly lower [20,25,31,32]. Moreover, quasi-isotropic laminates ([0/45/90]_s_) exhibit an intermediate behavior between [0] and [90] layups [33]. However, to the authors’ knowledge there has been no systematic investigation aimed at evaluating the influence of multidirectional laminate layup on the microstructural defects and mechanical response (i.e., tensile properties and failure modes) of CFRPA composites produced by FFF.

In addition, since composite materials offer great potential for optimization due to their orthotropic properties and multiple design variables (e.g., an infinite number of layups can be chosen), the adoption of predictive models to estimate the mechanical properties of 3D printed composites is of major importance. With this aim, various models can be considered and discussed. The rule of mixture (RoM) was applied by several authors [19,22,25,30,34] in the attempt to predict the properties of the printed parts reinforced with carbon fibers oriented at 0°, however significant deviations were reported for high fiber volume fractions. Therefore, Al Abadi et al. [35] and Yu et al. [26] applied the Volume Average Stiffness (VAS) method to predict the elastic properties of 3D printed CFRPA composites with different fiber content and infill patterns. The method involves a volume averaging of the stiffness matrices of the different regions of the fiber reinforced composites, namely shell, solid and infill. Both studies showed a good agreement between the predicted elastic moduli and Poisson’s ratio values and the experimental data [26,35]. Moreover, Choi et al. [36] demonstrated the validity of the Classical Laminate Theory (CLT) to model the elastic properties of highly oriented short carbon fiber reinforced composites produced using a common FFF printer. The results showed that CLT accurately predict the Young’s modulus and Poisson’s ratio for a wide range of laminate layups. Saeed et al. [33] used CLT to predict the mechanical behavior of CFRPA composites produced by the MarkForged^®^ FFF process. The results, in a similar fashion to those obtained by Polyzos et al. [37], are in good agreement with the experimental data for longitudinal, transverse and shear modulus as well as Poisson’s ratio. This confirms the potential of CLT to model FFF parts with continuous fibers.

The main limit of the aforementioned studies is that they are focused on elastic properties [26,33,35,36,37] and in some cases on longitudinal layup only [26,35]. In addition, the models were applied to parts reinforced with different types and volume fraction of fibers, contour and infill regions.

Within this context, this study is aimed at investigating different characteristics of longitudinal, cross-ply and quasi-isotropic [0/45/90/−45]_S_ and [0/±60]_S_ CFRPA composites produced by FFF process and characterized in term of microstructure (void content and distribution, other defects), mechanical properties (tensile modulus and strength), and failure morphology and mechanism. Moreover, different modeling approaches at micromechanical (i.e., RoM) and lamina level (i.e., VAS method and Classical Laminate Theory), were applied to predict the elastic modulus and tensile strength of the composites from raw filaments and lamina properties, respectively. The predicted mechanical properties were compared with experimental data obtained on laminates that differs only for the layup adopted. 

## 2. Materials and Methods

### 2.1. Materials and Printing Process

**Figure 1:**
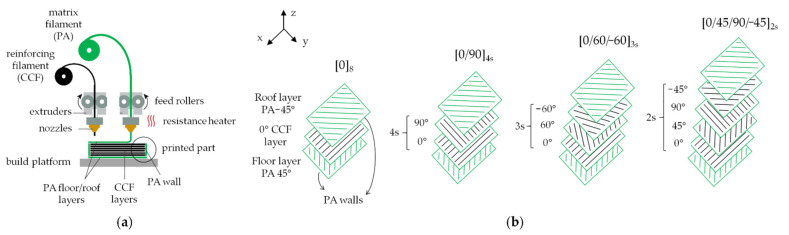
(**a**) Schematic representation of MarkForged^®^ FFF printing process; (**b**) internal structure of the CCF/PA composites comprising PA roof/top layers, CCF reinforced intermediate layers showing fibers infill with different orientations (0°, 90°, 45°, 60°) according to the layup adopted and a PA contour for each layer.

Continuous carbon fiber reinforced polyamide composite (CCF/PA) parts were produced using the Mark Two^®^ 3D printer from MarkForged^®^ (Watertown, MA, USA). This system is based on a patented dual extrusion FFF technology [38]. A schematic representation of the FFF process is illustrated in Figure 1a. The Mark Two is equipped with two extrusion nozzles that allows the printing of two spools of filaments, one of a plastic matrix and one of pre-impregnated continuous fibers, respectively (Figure 1a). Neat polyamide (referred as PA filament) and pre-impregnated continuous carbon fibers filaments (referred as CCF filament or tow) were chosen as plastic matrix and reinforcement, respectively. The raw materials were supplied by CMF Marelli Srl (Cinisello Balsamo, MI, Italy), official reseller of MarkForged^®^ in Italy. MarkForged^®^ declares that PA filament is constituted by a specially tuned polyamide 6 copolymer [38]. The CCF filament is made of multiple strands of continuous carbon fibers embedded in a semi-aromatic polyamide matrix [38]. Different studies reported that the fibers match the profile of Toray^®^ T300 standard modulus carbon fibers [22,39].

The printing process of CCF/PA parts consists of two stages, repeated according to a layer-by-layer strategy. The PA matrix is extruded first to create the contour. The reinforcing CCF filament is subsequently deposited within the same layer via a second nozzle to form strands of continuous fibers inside the part. The printing head moves in the xy plane according to the tool path created using the slicing software Eiger, designed by MarkForged^®^ (Watertown, MA, USA). Thereafter, the build platform lowers by a layer thickness along the z axis to allow the layer-wise parts production until completion. The design of the system allows the arrangement of the reinforcing materials as required. In fact, the orientation of the continuous fiber strands can be adjusted in each layer by changing the raster angle settings through Eiger interface. The infill pattern and density, the number of floor/roof layers and the number of walls can also be manually configured. The printed objects are consistent with the part shape, designed using a CAD software, as well as the pre-defined internal layout of fibers and matrix for each layer (Figure 1b). However, the Mark Two^®^ printer does not give the same flexibility for other process parameters. The extrusion temperatures are fixed at 270 °C and 255 °C for PA and CCF filament, respectively, and the build platform is not heated. The layer thickness is set at 125 μm. The extrusion speed of the reinforcing filament was estimated by previous studies at 15 mm/s [19,20].

### 2.2. Samples Production

CCF/PA composites were manufactured to evaluate the effect of laminate layup on the printed parts properties. Using Eiger software to control the fibers orientation, the CCF/PA samples were built with four different layups, namely longitudinal (referred as [0]), cross-ply (referred as [0,90]_s_) and quasi-isotropic [0/±60]_s_ and [0/45/90/−45]_s_. These laminates, selected among the layups employed in the composite industry, are balanced and symmetric to simplify the design as well as the modeling process. The [0/±60]_s_ and [0/45/90/−45]_s_ specimens can be considered as three-layer and four-layer quasi-isotropic laminates, respectively.

Figure 1b shows a schematic view of the printing patterns and layups employed in this study. Note that one PA layer on the bottom and on the top of the printed parts (referred to as floor and roof layers in Figure 1) were always used. The floor layer improves sample adhesion to the build platform and prevents the possibility of fibers rupturing during part removal. The roof layer was used to guarantee symmetry. The perimeter of each layer (referred to as walls in Figure 1) was also printed by using a PA filament to improve the dimensional accuracy and achieve better surface finishing. The other regions of the samples were filled using the CCF tow by choosing the “isotropic pattern” option in Eiger software provided by Markforged (Watertown, MA, USA)and by modifying the rasters orientations according to the laminate layup adopted (Figure 1b). 

For mechanical testing, CCF/PA rectangular specimens with constant cross section were produced according to the ASTM D3039 standard. Tabs made of aluminum (length of 56 mm and thickness of 1.5 mm) were bonded to both ends using a high strength epoxy adhesive (3M™ Scotch-Weld™ Epoxy Adhesive DP420) to prevent stress concentration during gripping. The aluminum tabs and the gripping regions of the samples were roughened with SiC paper (180 grit) to improve adhesive bonding. The dimensions of the tensile specimens are reported in Table 1. 

Four rectangular samples were fabricated for each CCF/PA layup. PA filament was dried at 80 °C for 8h before printing and it was stored in a moisture-sealed dry box during the building process.

### 2.3. Filaments and 3D Printed Samples Characterization

Differential Scanning Calorimetry (DSC) was employed to evaluate the thermodynamic properties of the printable filaments. Samples with a mass of 8.5 ± 1 mg were analyzed in N_2_ flow (50 mL/min) with a heating/cooling from 30 °C to 300 °C at a rate of 10 °C/min using Pyris 1 DSC equipment (PerkinElmer, Waltham, MA, USA). A second heating step was also performed to get additional information on the thermal transitions of the materials. Thermogravimetric analysis (TGA) of the filaments was performed using a TGA/SDTA851e thermal analyzer (Mettler Toledo, Columbus, OH, USA). Samples with a mass of 35 ± 5 mg were introduced in alumina crucibles and their thermal stability and composition was investigated in a temperature range from 25 °C to 1000 °C under inert atmosphere with a heating rate of 10 °C/min. The degradation onset and maximum weight loss rate temperatures and the volume fraction of fibers were measured from TGA representative curves. The density of the raw materials was measured through a gas pycnometer (Ultrapyc 5000, Anton Paar QuantaTec, Boynton Beach, FL, USA).

The elastic modulus, tensile strength and elongation at break of the printable filaments were characterized by means of tensile testing according to ASTM D638-14 and ASTM D4018-17 standards for single PA and CCF tows, respectively. Uniaxial tensile tests were carried out using a MTS Criterion Model 43 universal testing machine (MTS Systems Corporation, Eden Prairie, MN, USA) equipped with a 5 kN load cell. Samples of 75 mm length were cut from both filament spools. Single PA filaments were tested without tabs using a suitable set of grips to prevent slippage. The cross-head speed was set to 1 mm/min in the initial part of the test to accurately determine the elastic modulus and then was increased to 10 mm/s. In contrast, CCF filaments were stuck on cardboard supports with a 15 mm diameter hole using a high strength epoxy adhesive. The cardboard was connected to the grips of the testing machine and the lateral sides were cut. The cross-head speed was set to 1 mm/min. The cross-sectional area of both filaments was estimated from optical microscopy measurements of their diameters at several points along their length. Per each type of material, five samples were tested. The mechanical behaviors of the 3D printed CCF/PA composites were investigated by tensile testing in accordance with ASTM D3039 standard. The tests were conducted at room temperature at the cross-head speed of 1 mm/min. An extensometer with a 25 mm gauge length was used to acquire strain data. For measurement accuracy, four samples were tested for each layup configuration. 

The morphology and microstructure of the raw filaments and 3D-printed samples were studied by means of optical microscopy. For optical analysis, YZ cross-sections of the samples were cut using a Brilliant 220 precision cut-off machine (QATM Gmbh, Mammelzen, Germany) and carefully mounted in acrylic resin. Standard metallographic preparation methods were used to manually polish the samples for microscopy observations. The cross-sections were observed using a Leica DMI 5000 M optical microscope (Leica Microsystems GmbH, Wetzlar, Germany) to study the microstructure at the filaments and 3D printed samples level. In this way, it was possible to assess the quality of the printable filaments and to identify process-induced defects in the final parts. The optical images were analyzed using Image J^®^ software to measure relevant microstructural features (i.e., filament diameters, number and diameters of fibers within the CCF tow, void volume fraction, beads dimensions). The entire cross-section of the 3D printed specimens, obtained by stitching 40 consecutive micrographs, was analyzed to determine the void content. Thresholding was performed using the ISODATA algorithm.

The tensile fractured surfaces of the raw filaments and 3D-printed parts were observed though a Leica EZ4W stereo microscope (Leica Microsystems Gmbh, Wetzlar, Germany)**.** Moreover, a detailed analysis of the typical fracture surfaces resulting from tensile testing of the printed composites and cryo-fracturing of the CCF filament was performed by field-emission scanning electron microscopy (FESEM) using a ZEISS Merlin^®^ FESEM (Carl Zeiss Microscopy GmbH, Jena, Germany). Samples were sputter coated with platinum before FESEM characterization to avoid charging. The images were taken at a voltage of 3 kV and a working distance of 3.5 mm.

## 3. Modeling Approaches

In the modeling of composite materials, the volume fraction of matrix and fibers has been used for predicting elastic properties and the strength of composites using several models at micromechanical (fiber/matrix) or lamina level. In the present study, PA and CCF filaments were referred to as “effective matrix” and “effective fibers”, respectively. The typical internal structure of the 3D printed composite samples consists of different regions: roof and floor PA layers, PA walls regions and CCF reinforced layers with different bead orientations as a function of the laminate layup (Figure 1b). These regions have different mechanical behavior due to the adopted material. The PA and CCF filaments volume fractions can be calculated from the Equations (1) and (2):

(1) $$V_{m}=\frac{V_{\mathrm{PA}}}{V_{\mathrm{composite}}}\;$$

(2) $$V_{f}=\frac{V_{\mathrm{CCF}}}{V_{\mathrm{composite}}}\;$$

where V_PA_ and V_CCF_ denotes the volumes of the PA and CCF filaments, respectively (in cm^3^) that is consumed to produce a specimen, and V_composite_ is the total volume of the printed part. These data can be obtained from the 3D printer software for each sample and are summarized in Table 2. The reported values were used to predict the tensile properties of the laminated composites (see Section 4.3). The volume fractions of the fiber can be obtained by multiplying the CCF pre-impregnated filament volume fractions of each layup by the fiber content of the pre-impregnated filament itself (37.4%) obtained from experimental analysis (see Section 3.1).

The elastic modulus and tensile strength of the printed parts were evaluated using different models commonly employed to predict the properties of continuous fiber reinforced composites:

- Modified Rule of Mixture (MRoM);
- Volume Average Stiffness (VAS) method;
- Classical Laminate Theory (CLT).

The MRoM method was adopted to predict the stiffness and strength of the composites directly from single PA and CCF filaments mechanical properties. Table 3 presents the material properties of nylon and CCF reinforced laminae, adopted in VAS and CLT calculations. These material properties were provided from the literature [20,29]. In Table 3, one defines the direction parallel to the fiber axis, two corresponds to the direction perpendicular to the fiber axis, and twelve defines the in-plane shear. The subscripts T and C refer to tensile and compressive properties, respectively.

### 3.1. Modified Rule of Mixture (MRoM)

A simple approach based on the Rule of Mixtures (RoM) is described. The RoM was modified by taking into account the void content of the printed composites by adding a “porosity correction factor”. Moreover, the fiber orientation efficiency factor was used to consider the effect of the laminate layup configuration. Therefore, the elastic modulus and tensile strength of the composite can be determined from Equations (3) and (4), respectively:

(3) $$E_{c}{=(\eta }_{0}E_{f}V_{f}{+E}_{m}V_{m}{)(1-\varphi )}^{2}$$

(4) $$\sigma_{c}{=(\eta }_{0}\sigma_{f}V_{f}{+\sigma }_{m}V_{m}{)(1-\varphi )}^{2}$$

where V_f_ and V_m_ are the volume fraction of the CCF and PA filaments, respectively, while E and σ stand for elastic modulus and tensile strength for composites (i.e., E_c_ and σ_c_), CCF filament (i.e., E_f_ and σ_f_) and PA filament (i.e., E_m_ and σ_m_). The term (1 − φ)^2^ quantifies the effect of porosity (φ) giving stress concentrations in the material, as reported by studies on cellular materials [40] and fibers’ reinforced composites [19,41]. The fiber orientation efficiency factor η_0_ was obtained using the Krenchel approach:

(5) $$\eta_{0}=\sum a_{n}\cos^{4}\theta_{n}$$

where θ_n_ is the angle between a group of parallel fibers and the direction of the applied load and a_n_ is the fraction of fiber oriented at θ_n_ with respect to the total amount of fibers. Using Equation (5), η_o_ is 1 for unidirectional fibers aligned in the loading direction, 0.5 for [0/90]_s_ composite and 0.375 for the quasi-isotropic layups.

### 3.2. Volume Average Stiffness (VAS) Method

The Volume Average Stiffness method has been implemented to predict the elastic modulus of the 3D printed composites [26,35,42]. The VAS method is based on the assumption of continuity of strain. Since the laminates are non-homogeneous (i.e., matrix and fibers-rich areas, neat PA walls and shell layers, voids), this is a reasonable assumption from a mechanical viewpoint.

The VAS method consists of three subsequent steps. Firstly, the five elastic constants of the materials used to print the composites were defined at the lamina level (Table 3). In this way, the stiffness matrices of neat PA and fiber reinforced materials (Q_PA_ and Q_CCF,_ respectively) can be populated:

(6) $$Q_{\mathrm{PA}}=\left[\begin{array}{ccc} E/{(1-\upsilon }^{2}) & \upsilon E/{(1-\upsilon }^{2}) & 0\\ \upsilon E/{(1-\upsilon }^{2}) & E/{(1-\upsilon }^{2}) & 0\\ 0 & 0 & G \end{array}\right]$$

(7) $$Q_{\mathrm{CCF}}=\left[\begin{array}{ccc} E_{11}/{(1-\upsilon }_{12}\upsilon_{21}) & \upsilon_{21}E_{11}/{(1-\upsilon }_{12}\upsilon_{21}) & 0\\ \upsilon_{12}E_{22}/{(1-\upsilon }_{12}\upsilon_{21}) & E_{22}/{(1-\upsilon }_{12}\upsilon_{21}) & 0\\ 0 & 0 & G_{12} \end{array}\right]$$

where E, G and υ are the elastic moduli, shear modulus and Poisson’s ratio of the PA ply and E_11_, E_22_, G_12_, υ_12_ and υ_21_ are the elastic moduli, shear modulus and Poisson’s ratio of the CCF ply (Table 3). A coordinate system transformation is applied to rotate the stiffness matrix Q_CCF_ according to the orientation θ of the fibers reinforced plies in the laminate, by relating the principal material or local axes, referred as 1–2, to the laminate or global axes, referred as x-y:

(8) $${\bar{Q}}_{\mathrm{CCF}}{=\mathrm{TQ}}_{\mathrm{CCF}}T^{-1}$$

where ${\bar{Q}}_{\mathrm{CCF}}$ refers to the stiffness matrix of the fibers reinforced plies in the global coordinate system and the transformation matrix T is defined as follows:

(9) $$T=\left[\begin{array}{ccc} \cos^{2}\theta & \sin^{2}\theta & 2\sin\theta \cos\theta \\ \sin^{2}\theta & \cos^{2}\theta & -2\sin\theta \cos\theta \\ -\sin\theta \cos\theta & \sin\theta \cos\theta & \cos^{2}\theta -\sin^{2}\theta \end{array}\right]$$

For neat PA plies ${\bar{Q}}_{\mathrm{PA}}{=Q}_{\mathrm{PA}}$ as the material is perfectly isotropic (Table 3). Finally, the stiffness of the composite specimens is determined by volume averaging of the stiffness matrices of each material and each fiber reinforced lamina using Equation (10):

(10) $${\bar{Q}=Q}_{\mathrm{PA}}V_{m}+{\bar{Q}}_{\mathrm{CCF}}V_{f}$$

The elastic modulus E of the printed parts can be obtained from the ${\bar{S}}_{11}$ component of the reduced compliance matrix $\bar{S}$, that is the inversion of global stiffness matrix $\bar{Q}$ given in Equation (10):

(11) $$E=\frac{1}{{\bar{S}}_{11}}$$

The described formulation has been coded as a MATLAB script (MATLAB 2019a, The MathWorks, Natick, MA, USA) to compute the stiffness and compliance matrices of the composites with various layups.

### 3.3. Classical Laminate Theory (CLT)

Classical Laminate Theory (CLT) was applied to predict the macro-mechanical properties of the CCF/PA composites. The assumptions of the theory are reported in [43]. CLT allows us to calculate the stress-strain relations of orthotropic laminates consisting of stacked unidirectional plies of matrix or reinforced materials. The formulas used for CLT analysis are described below [43]. The stiffness matrix [Q]_k_ of each k ply can be populated using the five constants that determine the elastic response of a material, as described in Equations (6) and (7) for PA and CCF reinforced ply, respectively. The transformation matrix (Equation (9)) is used to rotate the stiffness matrix for various ply orientation according to Equation (8). The extensional [A], coupling [B] and bending [D] stiffness matrices can be evaluated by considering the position and thickness of each ply in the layup, as follows:

(12) $$A_{\mathrm{ij}}=\sum_{k=1}^{n}{\bar{Q}}_{k}(z_{k}{-z}_{k-1})$$

(13) $$B_{\mathrm{ij}}=\frac{1}{2}\sum_{k=1}^{n}{\bar{Q}}_{k}{(z}_{k}^{2}{-z}_{k-1}^{2})$$

(14) $$D_{\mathrm{ij}}=\frac{1}{3}\sum_{k=1}^{n}{\bar{Q}}_{k}{(z}_{k}^{3}{-z}_{k-1}^{3})$$

where z_k−1_ and z_k_ represents the distance of the bottom and top surface of the ply k from the laminate midplane, respectively. The midplane strains ε_0_ and curvatures κ can be calculated from the applied loads (i.e., normal stresses N and moments M) according to the Equation (15):

(15) $$\left\{\begin{array}{c} \varepsilon_{0}\\ \kappa \end{array}\right\}={\left[\begin{array}{cc} A & B\\ B & D \end{array}\right]}^{-1}\left\{\begin{array}{c} N\\ M \end{array}\right\}$$

For balanced and symmetric laminates, A_16_, A_26,_ A_61_, A_62,_ and the B_ij_ terms are equal to zero. As a result, the shear-extension and bending-extension coupling effects vanish. Moreover, in tensile uniaxial tests only normal stresses are applied to the material, thus Equation (16) reduces to

(16) $$\left\{\varepsilon_{0}\right\}={\left[A\right]}^{-1}\left\{N\right\}$$

The strain across the plate thickness can be obtained as:

(17) $${\left\{\begin{array}{c} \begin{array}{c} \varepsilon_{x}\\ \varepsilon_{y} \end{array}\\ \gamma_{\mathrm{xy}} \end{array}\right\}}_{k}=\left\{\begin{array}{c} \begin{array}{c} \varepsilon_{x}^{0}\\ \varepsilon_{y}^{0} \end{array}\\ \gamma_{\mathrm{xy}}^{0} \end{array}\right\}$$

Finally, the strains and stresses for each ply k in the principal material direction can be evaluated from the calculated strains by using the transformation matrix [T] and the stiffness matrix [Q]_k_ according to Equations (18) and (19):

(18) $${\left\{\begin{array}{c} \begin{array}{c} \varepsilon_{1}\\ \varepsilon_{2} \end{array}\\ \frac{1}{2}\gamma_{12} \end{array}\right\}}_{k}=\left[T\right]{\left\{\begin{array}{c} \begin{array}{c} \varepsilon_{x}\\ \varepsilon_{y} \end{array}\\ \frac{1}{2}\gamma_{\mathrm{xy}} \end{array}\right\}}_{k}$$

(19) $${\left\{\begin{array}{c} \begin{array}{c} \sigma_{1}\\ \sigma_{2} \end{array}\\ \tau_{12} \end{array}\right\}}_{k}={\left[\begin{array}{ccc} Q_{11} & Q_{12} & 0\\ Q_{12} & Q_{22} & 0\\ 0 & 0 & Q_{66} \end{array}\right]}_{k}{\left\{\begin{array}{c} \begin{array}{c} \varepsilon_{1}\\ \varepsilon_{2} \end{array}\\ \gamma_{12} \end{array}\right\}}_{k}$$

A laminate failure analysis simulation tool [44] was used for CLT calculations. The elastic modulus was obtained from the extensional stiffness matrix:

(20) $$E_{11}^{\mathrm{lam}}=\frac{A_{11}A_{22}{-A}_{12}^{2}}{A_{22}t}$$

where “t” is the thickness of the laminate (Table 1). A progressive ply failure analysis was performed to determine the strength of the composites according to the ply discount model. An axial tensile load N_x_ is applied to the laminate and the stresses in each ply k are calculated using CLT analysis. The Tsai-Hill criterion was used to detect ply failure. If failure does not occur, the load is gradually increased. Once a lamina has failed, the elastic constants are fully degraded (“full reduction/ply removal” in the simulation tool) and the stiffness matrices are recalculated. This means that the damaged ply or plies will not carry any loads that are transferred to the undamaged layers. This procedure is repeated until all plies have failed so that the last ply failure is determined. Typically, in progressive failure analysis all transverse plies break first, followed by angled (e.g., 45° or 60° fiber orientation) and longitudinal plies, respectively, as a result of a load increment. The strength can be obtained by dividing the last ply failure load by the actual thickness of the laminate. For [0/90]_s_ layup, only the transverse and shear elastic constants (E_2_ and G_12,_ respectively) were set to zero after first ply failure (“2 mode” as stiffness reduction method in the simulation tool). In fact, this hypothesis is more appropriate for the transverse matrix cracking that usually occurs in [0/90]_s_ laminates as the cracked plies can still bear loading, at least in tension failure, due to the fiber’s contribution [43,45,46]. 

Since the printing strategy involves the deposition of a wall around the perimeter of each layer, the ply can be treated as a two-part system, as described in [36]: the infill region composed of CCF filaments only (or ±45° nylon in the floor/roof layers) and the PA wall, which is equal to a 0° uniaxial part. Therefore, CLT is used for the infill region of the composite, while the contribution of PA wall was modelled using the rule of mixtures (RoM). The elastic modulus and tensile strength are obtained as follows:

(21) $$E_{c}{=E}_{11}^{\mathrm{lam}}{(1-V}_{\mathrm{walls}}{)+E}_{11}^{\mathrm{PA}}V_{\mathrm{walls}}$$

(22) $$\sigma_{c}{=\sigma }_{\mathrm{lam}}{(1-V}_{\mathrm{walls}}{)+\sigma }_{1t}^{\mathrm{PA}}V_{\mathrm{walls}}$$

where $E_{11}^{\mathrm{lam}}$ and σ_lam_ are the stiffness and strength of the laminate obtained from CLT calculations, $E_{11}^{\mathrm{PA}}$ and $\sigma_{1t}^{\mathrm{PA}}$ denotes the mechanical properties of the axially oriented PA sample (Table 3) and V_walls_ denotes the volume fraction of the PA walls region obtained with simple geometrical consideration using Equation (23):

(23) $$V_{\mathrm{walls}}=\frac{{\mathrm{PW}}_{\mathrm{walls}}N_{\mathrm{walls}}t_{\mathrm{layer}}N_{\mathrm{layer}}}{V_{\mathrm{composite}}}\;$$

where are P and V_composite_ are the perimeter (Table 1) and volume (Table 2) of the samples, respectively, t_layer_ is the layer thickness, N_layer_ is the total number of layers and N_walls_ is the number of PA walls. The width of the nylon walls (W_walls_) was measured using a Leica EZ4 stereo microscope and was equal to 0.75 mm.

## 4. Results and Discussion

### 4.1. Properties of the Printing Filaments

**Figure 2:**
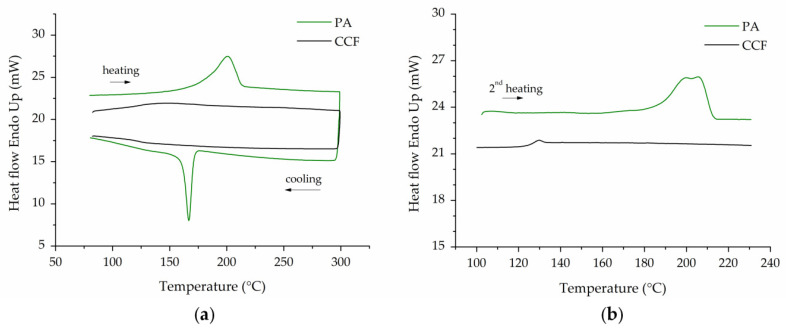
(**a**) DSC 1st heating–cooling curves; (**b**) DSC 2nd heating curves of PA and CCF filaments. The glass transition of CCF tow is clearly visible in the 2nd heating step.

Figure 2 illustrates the DSC thermograms of PA and CCF filaments. The first heating–cooling cycle is depicted in Figure 2a while the reheating step is showed in Figure 2b. 

For PA filament, the DSC curve displays melting and crystallization events at 201 °C and 167 °C, respectively (Figure 2a). These transition temperatures can be assigned to the α crystalline phase of PA6 [39]. In the second heating step, the thermal behavior of the PA filament slightly changes. In fact, the DSC trace shows a double melting peak with a shift to higher temperature values compared to the first thermal cycle (Figure 2b). This behavior is frequently observed for semi-crystalline polymers and can be attributed to melting–recrystallization–remelting mechanism or to the occurrence of two separate crystal morphologies [47]. The CCF filament exhibits the typical DSC curve of an amorphous material without melting or crystallization events (Figure 2a). The glass transition temperature (T_g_) is clearly visible in the reheating step around 127 °C (Figure 2).

The literature reports that the glass transition temperature of PA6I/6T copolymer, a semi-aromatic amorphous polyamide of the polyphthalamide (PPA) family, is between 125 and 127 °C [48]. This polymer, mentioned in MarkForged^®^ patent as the amorphous matrix of the CCF filament [38], shows better mechanical properties compared to linear semi-crystalline polyamides [48]. 

**Figure 3:**
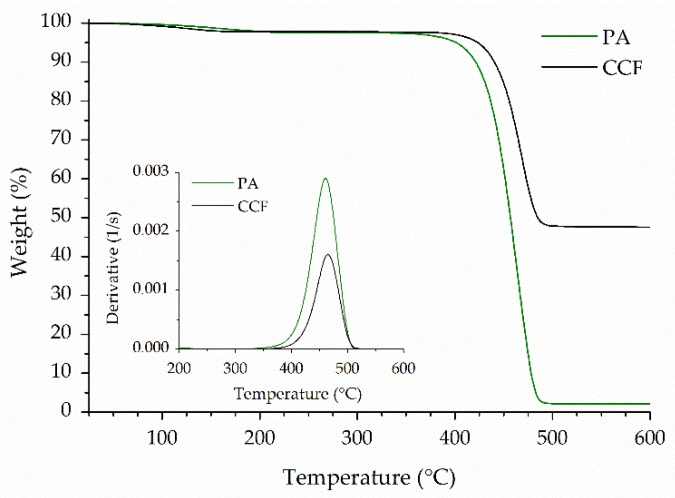
TGA curves in inert environment of PA and CCF filaments showing the different weight loss for the two materials. Insets of Figure 3: weight loss derivative curves that put in evidence the temperatures at maximum degradation rate (T_d peak_) of the filaments.

Figure 3 shows the weight loss (%) curves of neat PA and CCF filaments obtained by thermogravimetric analysis in inert environment. Derivative weight curves are also illustrated in the inset of the same figure. In the first part of the thermogram, a slight weight decrease (about 2%) is recorded for both filaments at temperatures between 100 °C and 160 °C (Figure 3). This event is related to the evaporation of moisture absorbed by polyamides at ambient temperature. The main degradation step, corresponding to the decomposition of the polymer chain backbone, starts at 404 °C and 421°C for PA and CCF filaments, respectively. The higher thermal stability observed for CCF tow could be attributed to the high amount of carbon fibers (Figure 3) and the different nature of the polyamides used in the filaments (Figure 2). The content of continuous carbon fibers in the CCF tow could be derived from the residual weight loss at 1000 °C (Figure 3), that correspond to 47.2 wt.%. 

Table 4 summarized the thermal and physical properties of the printable filaments. The volume fraction of fibers in the CCF tow was calculated using Equation (24):

(24) $$V_{\mathrm{fibers}}=\frac{W_{\mathrm{fiber}}\rho_{m}}{W_{\mathrm{fiber}}\rho_{m}{+W}_{m}\rho_{\mathrm{fiber}}}$$

where ρ_m_ and ρ_f_ are the density of the PPA matrix (1.18 g/cm^3^) [49] and Toray T300 carbon fibers (1.76 g/cm^3^) [50], respectively, and W_fiber_ is the weight fraction of fibers derived from TGA curve. Similar values of transition and degradation temperatures and fibers volume fraction were obtained by other authors [21,25,34,39].

**Figure 4:**
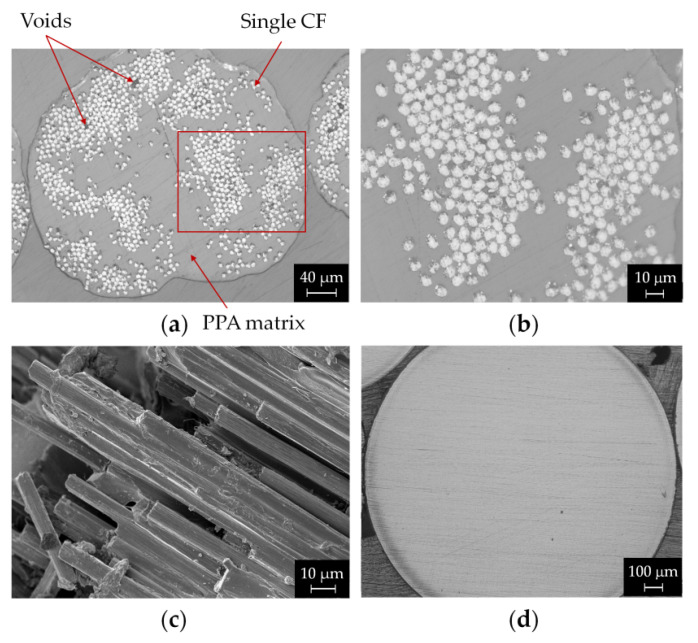
Optical images of polished cross-sections of (**a**) CCF filament and (**b**) magnified view of a high fibers concentration region of the CCF filament; (**c**) FESEM micrograph of CCF tow cryo-fractured surface and (**d**) cross section of PA as-received filament.

Microstructural analysis of PA and CCF filaments was performed using optical and electron microscopy (Figure 4). As received CCF filaments are composed of multiple strands of continuous carbon fibers embedded in an amorphous polyamide matrix (Figure 4a). The filament has an average diameter of 380 μm and contains around 1000 carbon fibers with an individual diameter of 7.1 μm. (Table 5)**.** Moreover, it is observed that carbon fibers are not uniformly distributed in the matrix phase and large areas which are rich in fibers can be seen. The inhomogeneous reinforcement distribution across the cross section is confirmed by the difference between the local fiber volume fraction in these regions (depicted in Figure 4b) and the average values obtained for the overall filament (Table 5). Matrix-free zones containing fibers that remain non-impregnated also appear in the as received filament. As a result, small voids can be distinguished in these areas (Figure 4a), thus leading to weaker interfacial bonding between the fiber and matrix. 

These defects can be attributed to the high melt viscosity of the PPA matrix that hinders the mobility of the polymer chains during filament fabrication by coextrusion [38]. To improve pre-impregnated filament quality, different approaches can be considered. Garofalo et al. [51] found that drawing the fibers over stationary convex pins helps the fiber tow to diverge, thus uniformly spreading before impregnation. Other strategies could be based on rheology (e.g., use of polymers with low melt viscosity, extruder geometries able to induce non-laminar melt flow, viscosity modifiers) or surface functionalization of carbon fibers to enhance wetting between fiber and matrix (e.g., acid treatments or oxidation) [38].

The SEM image in Figure 4c is a side-view of a cryo-fractured surface of the CCF filament showing numerous longitudinal continuous carbon fibers. PA filaments show a fully dense cross section with a nominal diameter of 1747 μm (Figure 4d).

**Figure 5:**
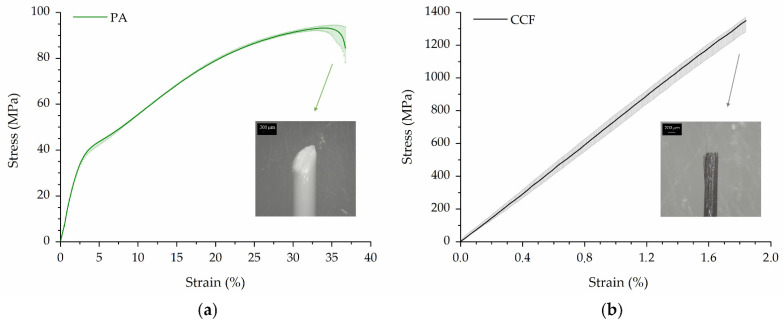
Tensile stress–strain behavior and fractured surface morphology of (**a**) PA and (**b**) CCF filaments.

The tensile stress–strain curves of single PA and CCF filaments are illustrated in Figure 5. The stress versus strain response of PA filaments highlighted the high ductility and elongation at failure of the neat polyamide 6. In detail, the curve could be separated in four regions: reversible linear elasticity at low stress, nonlinear elastic to viscoelastic transition, strain hardening and necking until rupture (Figure 5a). The observation of the fractured surface confirmed that the filament failed in a macroscopically ductile manner (inset of Figure 5a). By contrast, CCF filaments showed a linear elastic behavior until the rupture of the samples (Figure 5b). The elongation at break is relatively small due to the high content and brittle nature of the carbon fibers embedded in the filaments. However, its elastic modulus and tensile strength are significantly higher compared to pure PA filament (Table 6). Similar tensile properties and failure morphology of the CCF tows were also reported by Peng et al. [34]. 

### 4.2. Properties of the 3D Printed Composites 

#### 4.2.1. Microstructure and Voids

**Figure 6:**
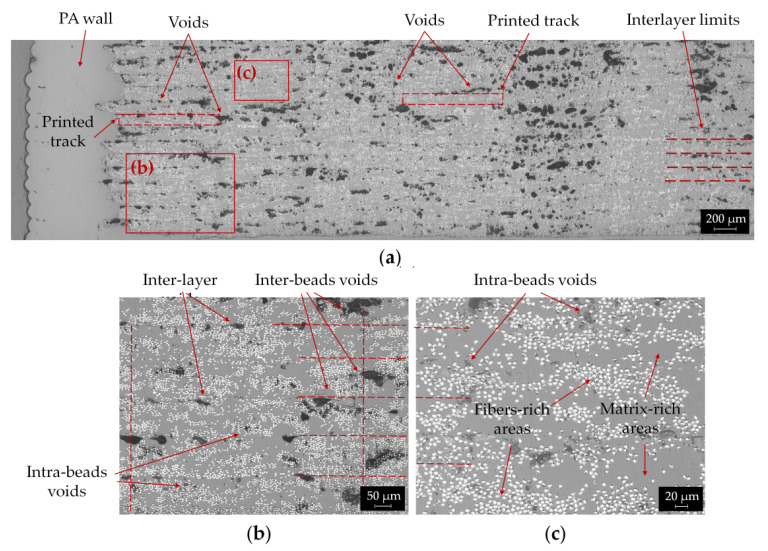
Cross-sectional optical micrographs of a longitudinal CCF/PA composites sample at various magnification levels: (**a**) panoramic view; (**b**) magnified view highlighting interlayer limits (horizontal dashed lines), beads interfaces (vertical dashed lines) and different types of voids (intra and inter layers); (**c**) intra-beads regions with alternated matrix-rich and fibers-rich areas.

Microstructural analysis of the printed parts was performed using optical microscopy. Images of YZ cross sections of CCF/PA samples with [0] layup at various magnification are presented in Figure 6. 

Figure 6a shows the cross section of longitudinal reinforced composites. The image clearly reveals the typical microstructure of the printed parts produced using the FFF process. Wall and infill of neat PA and CCF materials, respectively, can be distinguished along with the multiple stacked layers through the thickness of the sample. Large voids can also be observed in several regions of the printed part. A high pore content (11.1%) was calculated through ImageJ^®^ software (Bethesda, MD, USA). Similar values of porosity for the longitudinal composite were reported by other authors by means of optical microscopy [16,17,18] and micro-computer tomography techniques [25]. 

Figure 6b shows a magnified area of the entire cross section, where the interfaces between the layers are highlighted with horizontal red lines; in addition, the borders between adjacent printed beads (see vertical red dashed lines) are put in evidence in the image. The interlayer limits can be identified by the presence of nearly elongated voids, referred to as “inter-layer voids” in Figure 6b. The interface between adjacent beads is frequently delimited by large intra-layer pores with irregular shape (“inter-bead voids”) that represent almost 80% of the total porosity content (Figure 6a,b). However, the beads overlap is not uniform since the regions where the filaments join alternately exhibits more or less pores (Figure 6a,b). The dimensions of printed beads reveal that the filament experiences a considerable flattening during the printing process, from the tow diameter of 380 μm to a layer thickness of 125 μm and a width of 0.9 mm (i.e., equal to the diameter of the extrusion nozzle). Small round pores also appear in areas within the printed beads (“intra-bead voids”) (Figure 6b). These areas are shown in detail in Figure 6c. Within each printed bead, distinct regions of the matrix, fibers and voids can be observed. As a result, the composite parts revealed an uneven fibers distribution with polymer rich areas alternated to high fibers density regions (Figure 6c). The same microstructural features were found in [16,19,20], where the dimensions of the printed beads, the interlayer limits and the overlapping area between adjacent beads were described.

It is important to notice that in the CCF filament cross section large polymer rich areas also appear, although the amount of porosity is significantly lower compared to the printed parts (Figure 4a and Figure 6). This indicates that the FFF process is unable to completely promote adequate bonding between the printed beads, both layer by layer and side by side. In fact, the absence of pressure, the anisotropic thermal properties of the carbon fibers and the typical thermal history of the printing process prevent the coalescence of the contact surfaces between the beads, thus promoting the formation of inter-layer and inter-bead voids. Iragi et al. [20] found that almost three layers underneath the newly deposited filament keep experiencing heating above T_g_ of the amorphous PPA matrix (Table 4). However, the printed beads cooled rather quickly, thus hindering the molecular diffusion of the polymer chains across the contact surfaces. These heating/cooling cycles, which repeatedly occur during printing, strongly influence the bond quality between the adjacent beads, as well as the degree of interlayer consolidation. Regarding the “intra-bead voids”, some authors suggest that an incomplete matrix impregnation, possibly occurring in fibers rich areas of the as received filament (Figure 4b) and the relaxation of CF bundles during extrusion, could explain the presence of these small voids [19,20,25]. 

**Figure 7:**
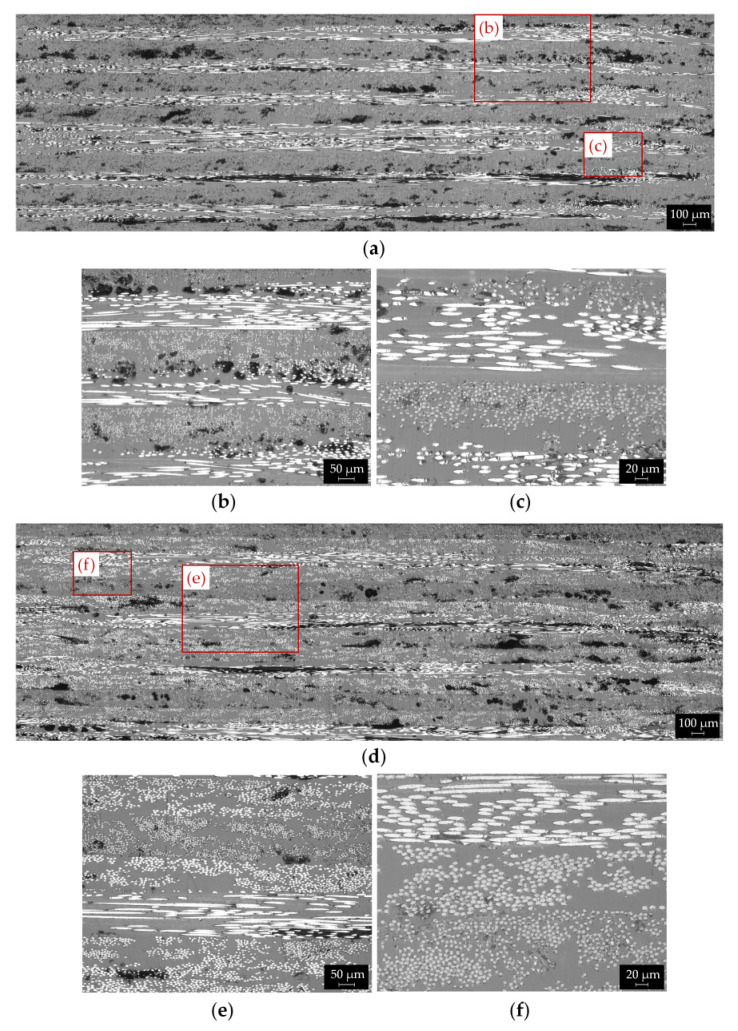
Optical micrographs showing typical YZ cross sections of (**a**) [0/90]_s_ and (**d**) quasi isotropic [0/45/90/−45]_s_ printed samples; magnified images in areas between stacked layers for (**b**) [0/90]_s_ and (**e**) [0/45/90/−45]_s_ parts highlighting interlayer voids and laminate layup sequence; magnified images in areas inside the printed beads for (**c**) [0/90]_s_ and (**f**) [0/45/90/−45]_s_ samples revealing uneven fibers distribution and matrix-rich areas.

Figure 7a,d show some panoramic micrographs of the YZ cross sections of [0/90]_s_ and [0/45/90/−45]_s_ printed parts, obtained by stitching together several 50x images.

Large void areas are clearly visible in these images. The porosity content is comparable to that observed in longitudinal composite and reaches values of 11.1%, 11.3% and 11.6% for the [0/90]_s_, [0/±60]_s_ and [0/45/90/−45]_s_ layups, respectively. However, the voids shape and distribution differ from that of the longitudinal laminates (Figure 6) as the porosities are more elongated (“crack-like void”) and are mainly located in the interlayer areas (Figure 7). These type of pores represent the largest portion of the voids for the cross-ply and quasi-isotropic composites.

The panoramic cross-sectional images are magnified in Figure 7b,c,e,f for [0/90]_s_, [0/45/90/−45]_s_ composites, respectively. The interlayer voids can be easily observed in the images, while the laminate layup sequence can be identified by the different cross-sectional shape of the fibers (Figure 7b,e). Moreover, the magnified micrographs of Figure 7c,g reveals regions with high fibers content alternated to large polymer-dominated areas, as already observed in the longitudinal sample. Smaller voids within the layers, probably related to incomplete sintering between beads or inherent porosity of the pre-impregnated feedstock filament, can also be noticed (Figure 7c,f).

It is important to outline that the void shape and distribution significantly change for the different laminate layup from irregular gaps in the inter-bead limits for [0] samples (Figure 6) to elongated voids located at the interface between layers for the other laminate layups (Figure 7). In fact, the laminate layup strongly influences the temperature profiles developed during the FFF process, as observed by Kousiatza et al. [52] through in-situ monitoring of the temperature variations during printing of CCF/PA composites. The authors found that longitudinal composites (0°) exhibited a uniform temperature profile with a gradual decreasing during the building process. By contrast, composites with a biaxial layup (±45°) displayed non-uniform temperature variations in one layer to the next one. Higher temperatures are recorded in the first layers, followed by a quick decreasing trend as the printing process progresses [52]. Therefore, it can be assumed that cross-ply and quasi-isotropic samples experience lower temperatures for a longer period during processing if compared with the longitudinal composites. This leads to the reduction in inter-diffusion efficiency of the polymer chains between consecutive layers. Consequently, large voids are formed in the interlaminar areas (Figure 7), resulting in poor adhesion. On the other hand, the smaller distance travelled by the printing head to deposit contiguous fiber bundles at an angle of 45°, 60° or 90° compared to 0° results in higher initial temperatures [52]. This promotes the softening of the adjacent bead once it is in contact with the newly deposited raster, thus leading to improved bonding and smaller interbeads voids compared to the longitudinal composites (Figure 6 and Figure 7).

Unfortunately, the above discussed defects are intrinsically associated to the FFF technique and the void content of the printed parts is significantly higher compared to laminates produced by conventional techniques (<1%) [53]. However, a method to improve the consolidation of the part is to perform a post-process treatment of hot compression molding. Several authors observed a reduction in void content between 50% [25,53] and 87% [54] by hot pressing the printed parts. As a result, a great improvement of mechanical properties was achieved [25,53,54]. On the other hand, this post-processing step requires the adoption of molds, which limits the main advantage of 3D printing (i.e., the ability to produce near-net-shape parts with complex geometry) and increases the manufacturing costs and time. Finally, it is worth noting that other approaches could be implemented to reduce the void content, such as printing inside at low pressure conditions inside a vacuum chamber [55], using a material preheating system [56], a layer post-compaction stage [57,58] or a combination of the two [59,60]. This last method is usually adopted in Automated Fiber Placement (AFP) to improve material consolidation [61].

#### 4.2.2. Tensile Properties and Fracture Surfaces

**Figure 8:**
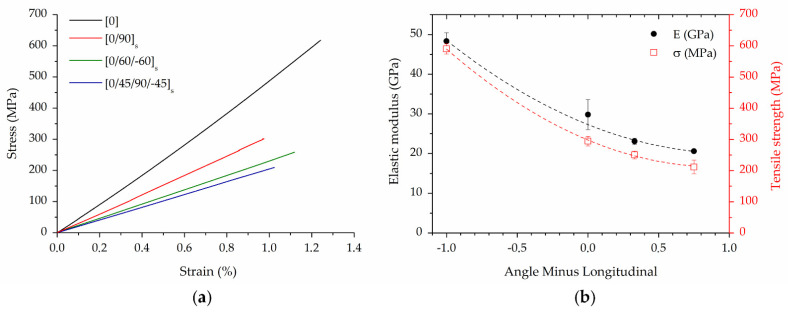
(**a**) Typical stress–strain curves of CCF/PA composites with various layups; (**b**) relationship between mechanical properties (elastic modulus and strength) and Angle Minus Longitudinal of the CCF/PA laminates.

The typical stress–strain curves of CCF/PA samples with different layups are reported in Figure 8a. The composites display a linear elastic behavior until failure (Figure 8a). This behavior is comparable to that observed in single CCF filaments (Figure 5b) and indicates that the fibers effectively withstand most of the applied stresses. Very low strain at break (in the range of 1–1.2%) was recorded, which is typical of brittle materials (Figure 8a). A slight stiffening effect was observed in the tensile curves as a result of fibers straightening under increasing load. This behavior was also reported in the literature for CFRPA composites produced by FFF [20].

The longitudinal laminate exhibits the highest load bearing capacity as the fibers are aligned in the tensile loading direction. As expected, the mechanical performances gradually decrease for the cross-ply and quasi-isotropic composites (Figure 8a). In fact, the laminate layup strongly affects the mechanical response of the material as fibers in angled plies could not well withstand the axial tensile loads. As shown in Figure 8b, the elastic modulus and ultimate tensile strength of the composites decrease with a quadratic function of the Angle Minus Longitudinal (AML). AML is the difference between the fraction of angled plies and the fraction of longitudinal (0°) plies [33]. This metric shows if a layup is dominated by off axis plies or not. Therefore, the four layup can be classified in terms of tensile properties as follows: longitudinal > cross-ply > quasi-isotropic [0/±60]_s_ > quasi-isotropic [0/45/90/−45]_s_. The elastic modulus declines by 38% and 57% for [0/90]_4s_ and [0/45/90/−45]_2s_ layup, respectively, compared to the longitudinal samples (Figure 8b). A high reduction in strength was also reported due to the low transverse and interlaminar properties of the composites (Figure 8b) [19,20,25]. 

The stiffness and strength in other orthotropic directions are also important to define the ply elastic and strength properties of a laminate. Several literature studies addressed in detail the mechanical behavior of additively manufactured PA/CCF composites with [90] and [±45] layups, thus determining their transverse and in-plane shear properties, respectively [20,31,32,33]. Laminates with [90] layup exhibit poor mechanical properties (E = 4.0 GPa and σ = 19 MPa) [32] and very low elongation break (ε = 0.5%) [20,31,32]. Brittle fracture in the plane perpendicular to the applied load and parallel to the fibers is commonly observed [20,32]. Angle-ply [±45] laminates shows low modulus (E = 2.3 GPa) and moderate fracture strength (σ = 90.4 MPa) [32]. However, the stress-strain response is non-linear and high plastic strains are achieved owing to the ductile nature of the polyamide matrix [20,31,32,33]. 

A comparison between the experimental results and these literature data is useful to better explain the mechanical behavior of the different layups. Longitudinal laminates provide the highest axial strength and stiffness (Figure 8a). Laminates with [±45] and [90] layup exhibit low strength and stiffness due to the mechanical response of the material depending on the matrix properties and manufacturing defects [20]. Cross-ply and quasi-isotropic laminates display an intermediate behavior between the longitudinal composite and the other layups. In fact, it has been found that the ratio between 0° plies and off-axis plies has a great role in determining the stiffness and strength of the laminate (Figure 8b). Moreover, these layups differ from those previously described as they are balanced and symmetric, resulting in nearly constant strength and stiffness regardless of the direction in which they are loaded. This quality is highly relevant for structural applications. Accordingly, cross-ply laminates are widely used in industry. However, if the loading condition are complex (i.e., off-axis bending and tension), quasi-isotropic composites offer superior performance.

**Figure 9:**
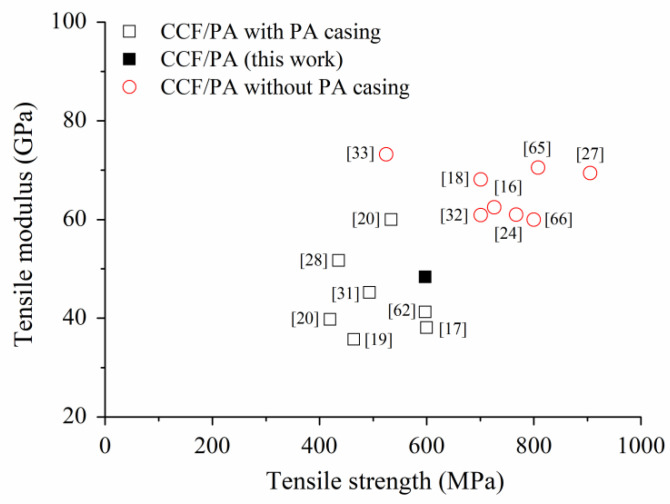
Comparison of the tensile properties of longitudinal CCF/PA laminates produced by the FFF process using MarkForged printers. The expression “PA casing” refers to the contour and outer layers of neat PA.

As reported in Table 2, the longitudinal laminate has 27 vol.% of carbon fibers. This layup reaches an average elastic modulus and strength of 48.3 GPa and 597.8 MPa. These values are comparable to those reported in the literature [17,19,21,22,31,62] for CFRPA composites produced by FFF with sandwich configuration (i.e., outer layers and walls of neat PA) and similar volume fraction of carbon fibers (Figure 9). Higher stiffness and strength were achieved in [18,20,25,32,33,63,64] by eliminating the neat PA contours and outer layers before testing (Figure 9), thus obtaining laminates with higher fibers content (up to 35 vol.%). It is worth noting that the CCF/PA composites exhibit a tremendous enhancement of the mechanical properties compared to those of the neat PA matrix [39]. The printed parts also show a great improvement (greater than 4x) of elastic modulus and strength with respect to short fiber reinforced polymer composites produced by FFF [12,65]. 

However, the tensile strength and in most cases the elastic modulus of CCF/PA composites produced by FFF are still lower compared to hot compression molded counterparts, irrespective of the layup adopted (Table 7). Moreover, it is worth noting that epoxy-based laminates currently used for primary load-bearing structures in aerospace applications are made by autoclave curing processes and exhibit significantly higher mechanical performances (E = 141 GPa and σ = 2205 MPa for HexPly^®^ 8552/AS4 unidirectional prepreg [66]). Poly (ether ether ketone) (PEEK)/carbon fibers laminates display better tensile properties too (E = 138 GPa and σ = 2070 MPa for Cytec APC-2/AS4 unidirectional prepreg [67]).

These differences can be explained by considering the current shortcomings of the MarkForged^®^ dual extrusion FFF process for continuous fiber reinforced laminates:

- high void contents, poor interlayer bonding and uneven fiber distribution due to the manufacturing process itself. In fact, the absence of pressure and the thermal conditions typical of the FFF process does not allow a proper thermo-mechanical consolidation between layers and adjacent filaments. These defects create stress concentration, thus causing the composite failure at lower stresses. This observation is confirmed by the experimental results reported in different studies, where a great enhancement of mechanical properties (22%, 36% and 145% for elastic modulus, tensile strength [25,73] and interlaminar shear strength [54], respectively) was achieved by reducing the void content via hot compression molding.
- low volume fraction of fibers (about 30%) compared to conventional laminates made from prepregs (>50%). In fact, the use of pre-impregnated filaments (those supplied by MarkForged® have 37 vol.% of continuous fibers) limits the fiber content in the printed parts and does not give the flexibility to modify it.
- low properties of raw materials. The polyamide matrix (i.e., PA6I/6T) used in the pre-impregnated filaments has lower mechanical performances compared to PEEK and epoxy resins. Moreover, low modulus carbon fibers (i.e., Toray T300) are used [18,21].
- process parameters cannot be modified by the user. For example, the extrusion temperatures are too low (270 °C and 255 °C for the neat polymer and pre-impregnated fiber filament, respectively) to print filaments based on high performance thermoplastics such as PEEK.

**Figure 10:**
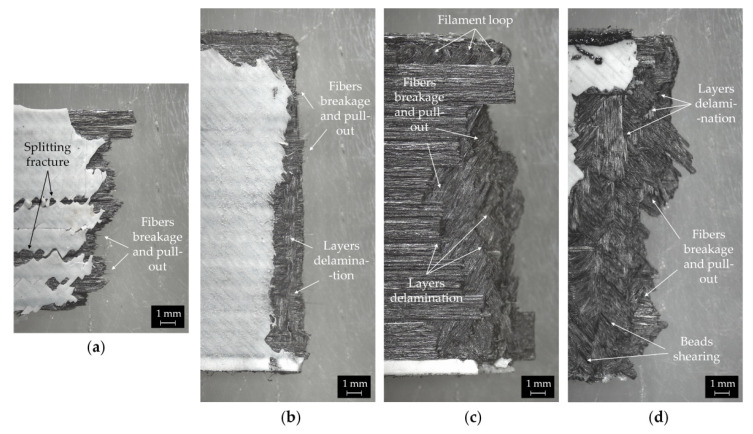
Macroscopic images of the fracture surface of (**a**) [0], (**b**) [0/90]_s_, (**c**) [0/±60]_s_ and (**d**) [0/45/90/−45]_s_ CCF/PA composites. Fiber breakage and pull-out, beads shearing and interlayer delamination are highlighted in the images.

Figure 10 shows the macroscopic images of the surface fracture of the printed composites, obtained by stitching four low magnification micrographs recorded using a stereomicroscope. From these micrographs, the different failure mechanism can be clearly detected. The longitudinal laminate fails with a step-like fracture mode without necking. Both vertical and horizontal cracks are observed indicating tensile rupture due to fibers breakage and pull-out (Figure 10a). This fracture mode is frequently reported for longitudinal fibers reinforced laminates produced both by traditional and additive technologies [17,32,70]. The fracture surface of the cross-ply composite is perpendicular to the tensile load direction and exhibits a relatively smooth profile (Figure 10b). The poor adhesion between 0° and 90° layers promotes nucleation of cracks and delamination (Figure 10b). The cracks propagate at the 0° layer with fiber rupture and pull-out until catastrophic failure (Figure 10b). The failure behaviors of the quasi-isotropic layups reflect the complex structure of these laminates. The fracture surfaces are very irregular (Figure 10c,d).

The rupture mode drastically changes to an interlaminar fracture (i.e., delamination between layers instead of fiber breakage). In fact, the weak intra- and interlayer bonding of the composites result in shear rupture between beads and delamination under mechanical loading (Figure 10c,d). Fiber breakage and pull-out can be also observed (Figure 10c,d). Moreover, filament loops on the top edge of the samples are clearly visible in Figure 10c. These loops are an inherent defect of the printing process, resulting from the sharp turn of the printed head at the perimeter of the part [16,19].

**Figure 11:**
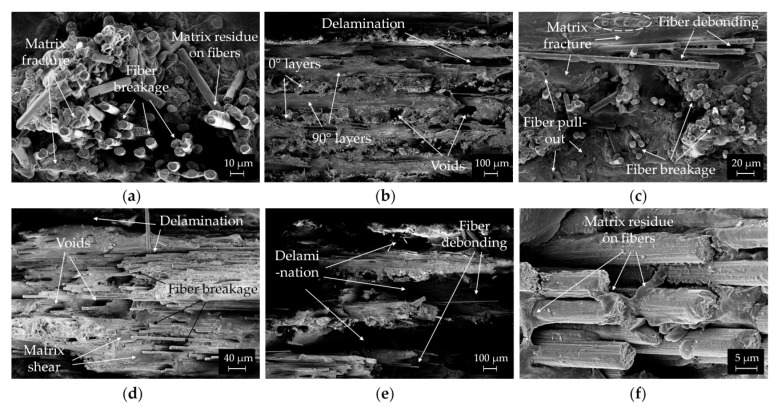
FESEM micrographs of the fracture surfaces of (**a**) [0], (**b**,**c**) [0/90]_s_, (**d**) [0/45/90/−45]_s_ and (**e**,**f**) [0/±60]_s_ CCF/PA composites. The mechanisms of fiber breakage, pull-out and debonding, matrix fracture and layers delamination are outlined in the images.

The micro-scale morphologies of the fracture surfaces of the CCF/PA composites were observed in detail by FESEM (Figure 11). 

Extensive fiber breakage can be noticed in the fracture surface of the longitudinal composites (Figure 11a), suggesting that the tensile failure mode is fiber dominant. This may indicate that the stresses are effectively transferred from the matrix to the fibers. Polymer residues on the surface of the pull-out fibers were also observed, revealing good fiber/matrix interfacial adhesion inside the beads (Figure 11a). Matrix fracture can be also detected in the FESEM image (Figure 11a). 

Figure 11b displays the fracture surface of [0/90]_s_ composites. The periodic and symmetric structure typical of the cross-ply laminates, with an alternating distribution of 0° and 90° oriented layers, can be easily detected. The fracture surface is relatively smooth. Few voids and delamination zones are visible at the interface between layers (Figure 11b). The different fracture behavior for 0° and 90° layers can be appreciated at higher magnification (Figure 11c). The longitudinal layers reveal an irregular fracture surface where most of the carbon fibers are embedded by the matrix material. A few pores created by fibers pull-out also appears after rupture (Figure 11c). The layer fails by fiber breakage and pull-out, indicating their ability of carrying high mechanical stresses before failure. By contrast, the fracture surface of the 90° layers is relatively smooth and shows localized matrix fracture zones, fibers breakage and extended debonding (Figure 11c). The debonded fibers have little polymer residue on the surface. Some micro-hills also appear in matrix rich areas (dashed circle in Figure 11c) as a result of the plastic deformation of the polymer.

FESEM micrographs of the tensile fractured surface of [0/45/90/−45]_s_ and [0/±60]_s_ composites are shown in Figure 11d,e. Large delamination zones in the interlayer areas, fiber debonding and matrix shear are present in both samples. These microscopic features could explain the macroscopic interlaminar fracture mode of the quasi-isotropic composites observed in Figure 10c,d. Moreover, matrix deformation in polymer rich regions, fiber breakage and few voids are clearly visible in the fractured surfaces (Figure 11d). At higher magnification (Figure 11f), protruding fibers coated by matrix material were also observed. The polymer adheres well on the fiber surface, indicating good interfacial bonding with the matrix in off axis plies too.

### 4.3. Comparison of Modeling Results with Experimental Data

The elastic modulus and tensile strength obtained with the previously described models are reported in Table 8, where predicted values are compared with experimental data from tensile tests. The relative error of the modelling results is obtained by:

(25) $$\mathrm{error}=\frac{\mathrm{prediction}-\mathrm{experimental}\;\mathrm{value}}{\mathrm{prediction}}100\%$$

One can see that the Modified Rule of Mixture, despite having the benefit of being simplistic and easy to use, is the less accurate method (Table 8). This behavior is coherent with other works [22,25,30,34], that show how the FFF-printed composites does not strictly behave according to the rule of mixture at high fibers volume fractions. A better agreement between experimental and predicted values was found for the elastic modulus by adding the term (1 − φ)^2^ to correct for porosity (Table 8). Nonetheless, the MRoM significantly overestimates the tensile strength of the composites (Table 8). In fact, the high void content and microstructure inhomogeneity of the CCF/PA samples promote cracks initiation and layer delamination, causing the samples to fail at lower stresses. Ultimately, the lower predictive accuracy of this approach is due to the fact that micromechanical models take into account the different orientation of the fibers inside each layer by using the fiber orientation efficiency factor (η_0_) only. The composite is treated as an isotropic material and the compliance matrix is reduced to one constant (see Equations (3) and (4)). In addition, the RoM considers as ideal the bonding between fibers and matrix.

Differently, VAS and CLT approaches take advantage of the exploitation of experimental values of the mechanical characteristics of the composite lamina. The elastic modulus values are found to be predicted with high accuracy using these methods with an error of 3.1–2.5 for [0], 0.9–2.3% for [0,90]_s_ and 2.6–4.4% for [0,45,90,−45]_s_ layups (Table 8). For [0, ±60]_s_ layup, the models slightly underestimate the elastic modulus (Table 8). The stiffness predictions are close to each other as the theoretical derivation of the models are similar. This means that using the RoM to adjust the CLT results for the effect of a printed PA wall is valid. For tensile strength, the predicted values by CLT approach are in good agreement with the tensile test results for the quasi-isotropic layups (Table 8). Higher discrepancies were found for the longitudinal and cross-ply laminates, however the modeling error is less than 9% (Table 8). 

Slightly higher modeling errors were found in the predictions of elastic modulus of longitudinal carbon fiber reinforced composites using the VAS method by Al Abadi et al. [35] (7.5%) and Yu et al. [26] (8.7%). The highest accuracy was reported in [33], where the Young modulus of longitudinal laminates was predicted with 0.4% error through CLT analysis performed using LAP software. However, the modeling error was significantly higher for the transverse modulus (15%) [33]. In addition, Polyzos et al. [37] found low error values (between 5 and 6%) in the predictions of the elastic properties of [0/90]_4s_, [0,45,90,−45]_2s_ and [±45]_4s_ by combining micromechanical and void models and CLT. However, it should be noted that these studies are solely focused on elastic properties.

The VAS method offers great flexibility in predicting the elastic modulus of 3D printed composites as different regions and materials (such as PA walls, PA roof/floor layers, fiber reinforced infill, etc.) can be easily accounted for in the model, as already found in previous literature [26,42].

Moreover, it is worth noting that CLT has other peculiar advantages. As it refers to a failure criterion, progressive failure analysis can be applied to the evaluate strength at break, damage accumulation and failure mechanism of a laminate. In fact, when a ply fails it is possible to determine how the composite will behave if a load is further applied. In addition, the response of the material to multi axial loading (such as those observed in real-life applications) can be calculated.

Therefore, it can be concluded that VAS and CLT methods provide an effective way to estimate the mechanical performance of 3D printed CFRPA composites with high fiber contents. These models can be employed by designers as predictive tools to tailor the fiber orientation of each layer of FFF printed CFRPA parts for specific stiffness and strength requirements.

## 5. Conclusions

In this research, the microstructure and tensile properties of 3D printed CCF/PA composites with various layups were investigated. In addition, the accuracy of different models for the prediction of stiffness and strength (i.e., Modified Rule of Mixture, Volume Average Stiffness method and Classical Laminate Theory), were compared. 

The conclusions can be linked to the objectives of the work as below:

1. Microstructural defects such as high voids content (values around 11%), inhomogeneous fiber distribution and poor bonding between layers were observed for all fabricated parts. These manufacturing defects are considered to be an intrinsic part of FFF techniques and can be attributed to an inadequate thermo-mechanical consolidation of the printed beads. However, the shape and the distribution of these voids depend on the adopted laminate layup: the non-homogeneous temperature fields generated during printing of cross-ply and quasi-isotropic samples promote the formation of large voids between layers, while the longitudinal composites exhibit porosities mainly in the inter-bead areas;
2. The mechanical performances are strongly affected by the laminate layup as well. The longitudinal composites offer higher stiffness and strength compared to cross-ply and quasi-isotropic composites due to the alignment of fiber in the loading direction, at the expense of strong mechanical anisotropy. It was also found that the mechanical properties of laminates gradually decrease as a function of the Angle Minus Longitudinal value. AML can be therefore used to determine the most suitable layup for specific loading conditions;
3. The investigation of the composite microstructure has proved to be particularly important in understanding the fracture mechanisms. In fact, process-induced defects and laminate layup significantly influence the failure modes that change from a step-like fracture dominated by fiber breakage and pull-out in the longitudinal composites to interlaminar failures for the quasi-isotropic layups;
4. VAS and CLT methods are effective in predicting the mechanical properties of 3D printed composites with continuous carbon fibers. In fact, the models’ predictions showed good agreement with the experimental data. In contrast, the MRoM has not exhibited the same accuracy and it fails to predict the tensile strength.

Overall, the results of this research indicate that microstructural and mechanical analysis and the integration between laminate design optimization by using predictive models and such a manufacturing technology could drive the application of these materials within several industries, where complex light-weight components with load-bearing functionality are required, such as the automotive and aerospace industries.

## Figures and Tables

**Table 1:** Sample notation, international standard adopted for tensile testing, laminate layup (fiber reinforced layers placed in between roof and top PA layers), and sample dimensions (length × width × thickness).

Sample Notation	ASTM Standard	Laminate Layup	Dimensions (mm)
[0]_s_	D3039	[45], [0]_8_, [−45]	250 × 15 × 1.25
[0/90]_s_	D3039	[45], [0/90]_4s_, [−45]	250 × 25 × 2.25
[0/45/90/−45]_s_	D3039	[45], [0/45/90/−45]_2s_, [−45]	250 × 25 × 2.25
[0/±60]_s_	D3039	[45], [0/60/−60]_3s_, [−45]	250 × 25 × 2.5

**Table 2:** Printing volumes and volume fractions of PA and CCF filaments regions in the CCF/PA composites obtained from Eiger software and Equations (1) and (2). The content of carbon fibers for each layup is also reported.

Laminate Layup	Sample Volume V_composite_ (cm^3^)	Volume of Printing Filaments (cm^3^)		PA Filament Volume Fraction V_m_ (%)	CCF Filament Volume Fraction V_f_ (%)	Fiber Volume Fraction (%)
		PA	CCF			
[0]_s_	4.42	1.26	3.16	28.5	71.5	26.7
[0/90]_s_	13.27	2.66	10.61	20.0	80.0	29.9
[0/45/90/−45]_s_	13.19	2.51	10.68	19.0	81.0	30.3
[0/±60]_s_	14.67	2.69	11.98	18.3	81.7	30.5

**Table 3:** Elastic constants and strength properties of neat PA [29] and CCF reinforced laminae [20] adopted for VAS and CLT modeling.

Sample Notation	PA Lamina	CCF Reinforced Lamina
Longitudinal elastic modulus—E_1_ (GPa)	0.94	69.4
Transverse elastic modulus—E_2_ (GPa)	0.94	3.5
In-plane shear modulus—G_12_, G_23_ (GPa)	0.34	1.9
Poisson’s ratio—ν_12_	0.4	0.41
Axial strength in tension—σ_1T_ (MPa)	53.8	905.3
Axial strength in compression—σ_1C_ (MPa)	53.8	426
Transverse strength in tension—σ_2T_ (MPa)	53.8	17.9
Transverse strength in compression—σ_2C_ (MPa)	53.8	66
Shear strength in tension—σ_1S_ (MPa)	68.9	43.4

**Table 4:** Thermal and physical properties of the raw filaments. Melting, crystallization and glass transition temperatures (T_m_, T_c_ and T_g,_ respectively) were determined by DSC. The glass transition of the PA filament was derived from [39]. Degradation temperatures (T_d onset_ and T_d peak_) and fibers volume fraction (V_fibers_) were evaluated from TGA curves.

Filament	T_m_ (°C)	T_c_ (°C)	T_g_ (°C)	T_d onset_ (°C)	T_d peak_ (°C)	V_fibers_ (%)	ρ (g/cm^3^)
PA	200.8	166.8	22.0	403.7	460.2	-	1.11
CCF	-	-	127.5	420.7	465.2	37.4	1.39

**Table 5:** Morphological properties obtained from image analysis of PA and CCF filament’s optical micrographs and calculated fibers number and volume fraction of a local region of the CCF tow with high fiber content (marked in red on Figure 4a). The filament diameters stated by MarkForged^®^ are reported in bracket.

Region	Morphological Property	PA	CCF
filament	Cross-sectional area (10^3^ µm^2^)	2402 ± 6	115 ± 1
	Diameter (µm)	1747 ± 4 (1750)	381 ± 5 (380)
	Porosity (%)	<0.1	1.3 ± 0.2
	Fibers diameter (µm)	-	7.1 ± 0.2
	Number of fibers	-	1032 ± 2
local	Measured area (10^3^ µm^2^)	-	4.65
	Number of fibers	-	104
	Fibers volume fraction (%)	-	87.3

**Table 6:** Mechanical properties of PA and CCF filaments. The value of the elastic modulus of the CCF tow was obtained from [34] as the extensometer could not be used during tensile test due to its low diameter (i.e., 380 µm).

Filament	Elastic Modulus (GPa)	Tensile Strength (MPa)	Elongation at Break (%)
PA	1.50 ± 0.99	93.9 ± 1.6	38.4 ± 3.0
CCF	74.43 ± 2.50	1344.0 ± 37.3	1.9 ± 0.1

**Table 7:** Mechanical properties and fibers volume fraction of longitudinal, cross-ply and quasi-isotropic CCF/PA composites produced by FFF and conventional polyamide based laminates manufactured by hot compression molding (HCM).

Laminate Layup	Elastic Modulus (GPa)	Tensile Strength (MPa)	Fiber Volume Fractions (%)	Reference
CCF/PA [0]	48.3	597.6	30	this work
	46.0	778.0	30	[68]
	83.2	939.7	35	[25]
	97.8	1322.6	42	[69]
	98.2	1308.9	42	[70]
CCF/PA [0/90]_s_	29.8	294.4	30	this work
	20.0	395.0	30	[68]
	35.4	408.0	40	[71]
CCF/PA [0/45/90/−45]_s_	20.6	211.6	30	this work
	13.0	232.0	30	[68]
	34.6	540.0	42	[72]

**Table 8:** Comparison between the elastic modulus and tensile strength of CCF/PA composites obtained experimentally and predicted using MRoM, VAS method and CLT analysis. The relative error (%) is reported in brackets.

Laminate Layup	Experimental		Modified Rule of Mixture (MRoM)		VAS Method	CLT Analysis	
	E (GPa)	σ (MPa)	E (GPa)	σ (MPa)	E (GPa)	E (GPa)	σ (MPa)
[0]_s_	48.3	597.8	55.2 (12.4%)	781.0 (23.4%)	49.9 (3.1%)	49.5 (2.5%)	651.1 (8.1%)
[0/90]_s_	29.8	294.4	30.9 (3.4%)	439.2 (33.0%)	29.5 (−0.9%)	30.5 (2.3%)	271.8 (−8.3%)
[0/±60]_s_	23.1	250.4	23.6 (2.2%)	337.7 (25.8%)	21.3 (−8.4%)	21.8 (−5.9%)	257.4 (2.7%)
[0/45/90/−45]_s_	20.6	211.6	23.2 (11.2%)	332.6 (36.4%)	21.1 (2.6%)	21.5 (4.4%)	211.3 (−0.1%)

## Data Availability

The datasets that support the findings of this study are available from the corresponding author upon request.
